# Human milk extracellular vesicles modulate inflammation and cell survival in intestinal and immune cells

**DOI:** 10.1038/s41390-024-03757-5

**Published:** 2024-11-28

**Authors:** Brett Vahkal, Illimar Altosaar, Ardeshir Ariana, Josie Jabbour, Falia Pantieras, Redaet Daniel, Éric Tremblay, Subash Sad, Jean-François Beaulieu, Marceline Côté, Emanuela Ferretti

**Affiliations:** 1 Department of Biochemistry, Microbiology and Immunology, University of Ottawa, Ottawa, ON Canada; 2 Centre for Infection, Immunity and Inflammation (CI3), University of Ottawa, Ottawa, ON Canada; 3 Children’s Hospital of Eastern Ontario Research Institute, Ottawa, ON Canada; 4 Department of Immunology and Cell Biology, Université de Sherbrooke, Sherbrooke, QC Canada; 5 Department of Pediatrics, Division of Neonatology, Children’s Hospital of Eastern Ontario, Ottawa, ON Canada

## Abstract

**Abstract:**

Human milk contains extracellular vesicles (EVs) that carry bioactive molecules such as microRNA, to the newborn intestine. The downstream effects of EV cargo on signaling and immune modulation may shield neonates against inflammatory diseases, including necrotizing enterocolitis. Premature infants are especially at risk, while human milk-feeding may offer protection. The effect of gestational-age specific term and preterm EVs from transitional human milk was characterized on human intestinal epithelial cells (HIECs and Caco-2), primary macrophages, and THP-1 monocytes. We hypothesized that term and preterm EVs differentially influence immune-related cytokines and cell death. We found that preterm EVs were enriched in CD14 surface marker, while both term and preterm EVs increased epidermal growth factor secretion. Following inflammatory stimuli, only term EVs inhibited secretion of IL-6 in HIECs, and reduced expression of pro-inflammatory cytokine IL-1β in macrophages. Term and preterm EVs inhibited secretion of IL-1β and reduced inflammasome related cell death. We proposed that human milk EVs regulate immune-related signaling via their conserved microRNA cargo, which could promote tolerance and a homeostatic immune response. These findings provide basis for further studies into potential therapeutic supplementation with EVs in vulnerable newborn populations by considering functional, gestational age-specific effects.

**Impact:**

- This study reveals distinct functional differences between term and preterm transitional human milk extracellular vesicles (EVs) highlighting the importance of gestational age in their bioactivity.
- Term EVs uniquely inhibited IL-6 secretion, IL-1β expression, and apoptosis following inflammatory stimuli.
- Both term and preterm human milk EVs reduced IL-1β secretion and inflammasome-induced cell death.
- Conserved human milk extracellular vesicle microRNA cargo could be a mediator of the anti-inflammatory effects, particularly targeting cytokine production, the inflammasome, and programmed cell death.
- These findings underscore the importance of considering gestational age in future research exploring the therapeutic potential of human milk extracellular vesicles to prevent or treat intestinal inflammatory diseases in neonates.

## Introduction

Human milk (HM) feeding has been shown to safeguard against infant inflammatory gut diseases, including necrotizing enterocolitis (NEC), which disproportionately affects premature neonates.^1^ The mechanisms underlying this protection remain poorly understood.^2^ The vulnerability of neonates to NEC is partially due to an inefficient response to T-cell-dependent antigens, weak T-helper 1 cell and antibody responses, and an impaired innate immunity. As a result, neonates have a high mortality rate under increased pathogen exposure.^3^ HM-contained extracellular vesicles (EVs) have been proposed as potential protective shields. Recent studies in immune cells have indicated a crucial role for HM EVs in immunoregulation.^4,5^

In a previous study, CD4 + T cell activation was inhibited in response to treatment with HM EVs.^4^ This transient suppression may be crucial for preventing excessive immune activation, thus promoting tolerance to foreign antigens.^4^ In a separate study, macrophages were shown to take up milk EVs and could have altered growth and proliferation as a result of treatment.^6^

In neonates, macrophages are present in mid-gestation^7,8^ and may contribute to the cytokine storm commonly seen in NEC.^9,10^ Neonatal sepsis and NEC are characterized by macrophage infiltration and secretion of inflammatory cytokines.^11,12^ A dose-dependent increase in pro-inflammatory cytokine expression was recently demonstrated in porcine macrophages following treatment with goat milk EVs,^13^ while protection against inflammation was seen in murine macrophages stimulated with bovine milk EVs.^5^ Additional findings of HM EVs being bioavailable, and bovine milk EVs restoring epithelial and immune cell barriers in the intestine of C57BL/6J mice,^5^ support the prospect of HM EVs regulating immune responses in breastfed neonates.

While controlling cytokine responses is important for the newborn’s immune system, regulation of immune cell activation and death is also critical. Overwhelming sepsis, a potential critical endpoint for NEC,^10^ may be mediated by pyroptosis, a programmed cell death mechanism that is induced by activation of the inflammasome.^14^ The inflammasome-induced pyroptotic cell death is partially responsible for maintaining intestinal microbial homeostasis and is tightly controlled. In response to danger signals, such as pathogenic bacteria and its outer membrane component lipopolysaccharide (LPS), pro-inflammatory cytokine IL-1β, along with other cytokines, is upregulated in both antigen-presenting and epithelial barrier cells.^15^ While multiple inflammasome blocking strategies have been trialed in animal models, inhibition of IL-1β and IL-18, two markers of pyroptosis, have conferred protection against sepsis.^15,16^

The timing of infant’s birth at term (>37 weeks of gestation) or preterm (<37 weeks of gestation)^17^ may influence the composition of mother’s milk and EV cargo within.^18–20^ The composition of mothers’ milk can vary further based on the stage of lactation, which can be divided into colostrum, expressed within the first 72 h post birth; transitional milk, which is secreted within the first two weeks and up to one-month post-birth; and mature milk, which is expressed from one-month post-birth.^21^ We, and others, have reported significant differences in the HM protein, lipid, and microRNA (miR) levels,^22–26^ while preterm HM EVs have been found to have a greater impact on cell proliferation and migration in intestinal injury compared to term EVs.^22^ However, only a limited number of studies have investigated the effects of gestational age-specific EV preparations on human immune cells or animal models of NEC and characterized the miRs or proteins contained within these EVs,^22,24^ highlighting the need for further research in the context of intestinal inflammation.

To investigate the potential for gestational-age specific transitional HM EVs to regulate immune response, the impact on cytokine expression, secretion, and cell death were characterized. We hypothesized that the inflammatory response in human gut epithelial cells, primary macrophages, and in a leukemia monocytic cell line, THP-1 cells, differs following pre-treatment with term or preterm HM EVs. First, surface markers of term and preterm EVs were characterized. Then, cells were treated with HM EVs prior to inflammatory activation with heat-killed bacteria, or a low dose of LPS. We measured differential regulation of several key cytokines, while both term and preterm HM EVs reduced secretion of IL-1β in macrophages. We proposed HM EV miR cargo as an effector, wherein the most abundant miRs were conserved between donors. These findings could have implications for improved nutrient supplementation for vulnerable neonates and support the development of novel therapeutic interventions for intestinal inflammatory diseases.

## Methods

### Ethics

HM was obtained from donors following ethics approval by the University of Ottawa, The Ottawa Hospital, and the Children’s Hospital of Eastern Ontario (Research Ethics Board approval #H-03-20-5643). Donors were excluded if they had delivered via caesarean section, used antibiotics, or had active maternal genetic, immune, or chronic inflammatory diseases. Upon receiving written consent, donors sterilised their breast with an antibacterial wipe, followed by manual expression of 20 mL of milk into sterile containers. The HM samples were collected from 45 donors, and further categorized based on gestational age (GA) as either term (GA > 37 weeks) or preterm (GA < 37 weeks) (Table S1).

### Isolation of EVs

To obtain an EV-enriched pellet from freshly collected HM, we followed previously characterized protocols utilizing differential and ultracentrifugation.^27–32^ HM was centrifuged twice at 4600 × *g* for 30 min within 30 min of milk collection, to separate fat, cells, and cell debris. To reduce remaining fat content and remove larger apoptotic vesicles, the skimmed milk was then centrifuged at 20,000 × *g* for 30 min at 4 °C. The supernatant was carefully removed and ultracentrifuged twice at 100,000 × *g* for 1.5 h at 4 °C using a Beckman Coulter ultracentrifuge (Optima XPN-100 or Optima L-100 XP, Beckman Coulter) with a fixed angle rotor (Type 70 Ti, k factor: 216, Beckman Coulter). The EV-enriched pellet was resuspended in 400 μL of sterile phosphate-buffered saline (PBS) in aliquots and stored at −80 °C until further analyses.

### Super-resolution microscopy

EVs were immunolabeled and visualized using the EV Profiler Kit (ONI, Cat. No. EV-MAN-1.0) through direct stochastic optical reconstruction microscopy (dSTORM). Sample preparation and imaging were performed by Oxford Nanoimaging (ONI). Briefly, EVs were immobilized on microfluidic chips, followed by sample preparation according to kit instructions. The antibodies used included CD9/81-CF647 (kit, excitation/emission: 642/662 nm), CD63-CF568 (kit, excitation/emission: 562/583 nm), and CD14-BV421 (Cat No. 563743, BD Biosciences, excitation/emission: 405/421 nm). dSTORM imaging buffer was added prior to image acquisition. Imaging was conducted on the Nanoimager S Mark III microscope (ONI, United Kingdom) with 30 ms exposure. Surface markers were imaged with 640 nm, 561 nm, and 405 nm lasers at power settings of 30%, 50%, and 100% respectively, capturing 1000 frames per channel with an illumination angle of 47°. For subpopulation analyses of EVs expressing one, two, or three markers, ONI’s CODI online platform (https://alto.codi.bio) was utilized. This included density-based clustering analysis with drift correction and filtering to assess each vesicle.

### Nanoparticle tracking analysis

Extracellular vesicles were characterized using nanoparticle tracking analysis on a ZetaView PMX110 instrument (Particle Metrix, Germany). Samples were diluted in PBS and analyzed after calibration with 105 and 500 nm-sized polystyrene beads. The instrument was set for 85 sensitivity, 30 frames per second, and 100 shutter speed. ZetaView software was used to analyze the samples at 11 camera positions, and a system temperature of ~21 °C.

### Surface marker analysis

Term and preterm EVs were prepared for the overnight protocol following manufacturer’s instructions (MACSPlex Exosome Kit, Cat. No. 130-108-813, Miltenyi Biotec, Bergisch Gladbach, Germany). Briefly, 10 μg/mL of EVs, 120 μL of capture beads, or negative control buffer, were incubated with 15 μL MACSPlex EV Capture Beads in low protein binding tubes. The next day, 500 μL of MACSPlex Buffer was added to each tube and centrifuged at room temperature at 3000 × *g* for 5 min. Detection cocktail (CD9, CD63, CD81) was added to each tube and incubated at room temperature for 1 h. After washing, the samples were resuspended in a final volume of 150 μL. Samples were immediately analyzed on LSR Fortessa (BD Biosciences, San Jose, CA) following set-up instructions provided by MACSPlex Exosome Kit protocol. Following acquisition, samples were first gated using FlowJo software, version 10 (BD Biosciences) following kit instructions and a previously published protocol.^33^ Then further analyzed using MPAPASS software, whereby fluorescence intensity was normalized to background, as formerly described.^33,34^

To detect changes in Caco-2 CD14 surface expression, confluent cells were stained with anti-CD14 (BV421-conjugated, Cat. No. 563743, BD Biosciences), or unstained controls, and acquired on LSR Fortessa by gating on single cell populations based on forward and side scatter profile.

### Cell culture

Human intestinal epithelial cell line Caco-2/15 and human fetal small intestinal cells (HIECs) were cultured following established protocols.^35^ Briefly, the cells were cultured at 37 °C, 5% CO_2_-95% air, in Dulbecco’s modified Eagle’s medium (DMEM) supplemented with 10% fetal bovine serum (Gibco, Thermo Fisher Scientific, Waltham, MA). Caco-2 cells were grown to confluence for enterocytic differentiation, achieved 25 to 30 days post seeding, which has been previously described.^36^ Prior to the start of the treatments, cell culture dishes were matched for cell density, standardized previously. The density of Caco-2 cells was 750,000, and 300,000 for HIECs, per 35 mm dish.^36–38^ Both Caco-2 and HIECs were treated with term or preterm HM EVs (20 µg/mL) for 22 h, followed by heat-killed bacteria (*Escherichia coli* and *Salmonella typhimurium*) at a concentration of 10^9^ CFU/mL for 2 h.

Human peripheral blood mononuclear cells (PBMCs) isolation was performed using SepMate™ tubes (Cat. No. 85450, STEMCELL Technologies, Vancouver, Canada) and a density gradient centrifugation method with Lymphoprep™ (Cat. No. 07801, STEMCELL Technologies), following the manufacturer’s protocol. Monocytes were then isolated from PBMCs using negative selection with EasySep™ Human Monocyte Isolation Kit (STEMCELL Technologies). Briefly, the PBMCs were diluted to a concentration of 5 × 10^7^ cells/ml. The cells were then incubated with 50 µl/ml antibody isolation cocktail. Following addition of 50 µl of magnetic beads, cells were placed in a magnet for 10 min to capture non-monocyte populations. Monocytes isolated in the supernatant were counted and 10^7^ cells were added into a polystyrene petri dish (100 mm × 15 mm) pre-coated with recombinant human M-CSF (Cat. No. 216-MC, R&D Systems) to a final concentration of 10 ng/mL. The cells were then cultured in RPMI medium supplemented with 10% FBS (10 mL total volume per dish) for a period of 6 days at 37 °C in a humidified atmosphere containing 5% CO_2_ to promote macrophage differentiation. On day six, once fully differentiated, 1.5 × 10^5^ macrophages per well were seeded in 48-well plates and treated with term or preterm HM EVs (40 µg/mL) for 6 h, followed by LPS treatment (1 ng/mL) for 2 h.

THP-1 monocytes were obtained from ATCC (TIB-202™) and cultured in RPMI 1640 medium containing 10% FBS, 50 μg/mL gentamicin, and 1% β-mercaptoethanol. For microscopy, THP-1 cells were differentiated to macrophages by treatment with 50 ng/mL phorbol 12-myristate 13-acetate (PMA) for a duration of 72 h. Following differentiation, the cells were washed with PBS to remove residual PMA and cultured for an additional 24 h in PMA-free RPMI 1640 medium supplemented with 10% FBS. Cell death was induced following previously established protocols. First, THP-1 monocytes were treated with term or preterm HM EVs (40 µg/mL) for 6 h. To induce pyroptosis,^39^ cells were treated with LPS (1 ng/mL, Cat. No. L4524, MilliporeSigma, Burlington, MA) for 2 h, followed by nigericin (10 µg/mL, Cat. No. N7143-5MG, MilliporeSigma, Burlington, MA) for 1 h. For apoptosis,^40^ cells were treated with either emricasan (10 µM, Cat. No. S7775, Selleck Chemicals, Houston, TX) for 30 min, followed by etoposide (30 µM), or etoposide alone for 3.5 h.

### Cell viability

For a viablity assay of Caco-2 cells, 300,000 cells per well were seeded in 6-well plates and grown to confluence over four days. Then, 20 or 40 μg/mL of HM EVs were added to cells. Cell viability was measured after 6- or 24-h incubation using XTT Cell Proliferation Assay Kit (Cat. No. 10010200, Cayman Chemical, Ann Arbor, MI). Briefly, XTT reagent was thawed at room temperature. Then, equal amounts of reagents were mixed and 20 μL of total reagent was added per well in the dark. Following a 2-h incubation, absorbance at 450 nm was measured using Synergy H1 Multi-Mode Plate Reader (BioTek, Winooski, VT).

To assess the viability of THP-1 monocytes at the endpoint of all treatments, cells were stained for Annexin V and propidium iodide (Annexin V-FITC Apoptosis Staining/Detection Kit, Cat. No. ab14085, Abcam), according to kit protocol. The percentage of dead or alive cells was measured immediately using flow cytometry on BD Fortessa, followed by gating using FlowJo. Data from a total of 10,000 cells per sample were acquired.

### Microscopy

1 × 10^3^ macrophages per well were seeded in untreated µ-Slide 8 Well plates (Cat. No. 80826, Ibidi, Germany) prior to treatment with 20 µg/mL of DiR-labeled HM EVs or DiR-PBS background control. Briefly, 5 µL of XenoLight® DiR (Cat. No. 125964, Perkin Elmer, Waltham, MA) was added to 40 µL of EVs, and diluted to a final volume of 1 mL with PBS. Control samples included dye in PBS alone. EVs and negative control were stained for 40 min at room temperature on a rotating spinner, then ultra-centrifuged at 100,000 × *g* for 30 min to pellet vesicles. Prior to macrophage treatments, stained EVs and negative control were resuspended in 40 µL of PBS. To visualize cellular compartments, macrophages were further treated with 10 kDa dextran (FITC, 0.5 mg/mL, Cat. No. D1820, Invitrogen, Waltham, MA) for 1 h, and wheat germ agglutinin (WGA, AF350, 50 µg/mL, Cat. No. W11263, Thermo Fisher Scientific, Waltham, MA) for 30 min. Following treatment, cells were washed twice with cold PBS before proceeding to live-cell imaging or fixed in 4% PFA for confocal imaging. Live cell imaging was done on a Quorum Spinning Disk with a 63X objective (1.4 NA). Fixed cell imaging was done on a Zeiss LSM 880 with a 63X objective (1.4 NA). Image processing and background correction was performed using ImageJ.

### RNA extraction and quantitative real-time PCR (qPCR) analysis

At the end point of intestinal cell and macrophage treatments RNA was extracted using Qiagen RNeasy mini kit (Cat. No. 74104, Qiagen, Germany), following manufacturer’s instructions, and stored at –80 °C. cDNA was synthesized from 200 to 500 ng of total RNA using iScript™ cDNA Synthesis Kit (Bio-Rad, Hercules, CA) in a final reaction volume of 40 μl. Following synthesis, the cDNA was diluted to 10 ng RNA/μl and stored at –20 °C. qPCR was performed using SsoAdvanced Universal SYBR Green Master Mix (Bio-Rad) in a total reaction volume of 10 μl. CFX Connect thermocycler (Bio-Rad) was used for amplification of the target genes by a standard cycling protocol with an annealing/extension temperature of 60 °C. Gene expression across different treatments was analyzed using the comparative threshold cycle (Ct) ΔΔCt method reported previously.^41,42^ Reference genes *RPLO, PPIA*, and *B2M* were used for normalization based on published primer sequences, which have been shown to exhibit stable expression across epithelial or immune cells. All primer sequences are listed in Table S2.

### Cytokine secretion

The cell culture supernatants were collected following completion of treatments on intestinal cells and macrophages, and stored at –80 °C until analysis. Cell media was analyzed using ELISA for secretion of EGF, sCD14, IL-6, IL-8, or IL-1β at the end point of treatments. To determine secretion concentration, the ‘Quantikine human’ (R&D Systems, Minneapolis, MN) ELISA assay kits were used for individual cytokines. Delta (450–570 nm) absorbance was measured using Synergy H1Multi-Mode Plate Reader (BioTek). For primary macrophages, Human Inflammatory Cytokine Cytometric Bead Array (Cat. No. 551811, BD Biosciences) was used following manufacturer’s protocol. Data was acquired using LTR Fortessa (BD Biosciences) based on manufacturer’s set-up template. Standard curves and analyte concentrations were determined using BD CellQuest Pro software (BD Biosciences).

### Bioinformatics analyses

List of miRs with the highest abundance common to term and preterm HM EVs with their corresponding abundance values (counts per million, CPM) were uploaded for Ingenuity Pathway Analysis (IPA, Qiagen). For analysis of miR targets, cut-off filters included experimental and high-confidence predictions only. The predicted target genes were filtered to include targeted pathways related to apoptosis, inflammasomes, JAK/STAT signaling, MAPK signaling, Toll-like receptor cascades, necroptosis, pyroptosis, and other signaling pathways involved in immune responses, cell death, and disease processes. Input data and exact filtering parameters are listed in the supplementary materials (Table S3 and Fig. S3).

### Statistical analyses

Statistical analyses were conducted using GraphPad Prism 9 software (Version 9.0.0, GraphPad, San Diego, CA). Results were presented as mean ± SEM. Welch’s *t*-test was employed to compare two groups when comparing surface marker expression levels. When comparing three or more groups, a one-way analysis of variance (ANOVA) was performed. Multiple comparison tests were used post-hoc to identify specific group differences following a significant ANOVA result. Tukey’s test was performed to compare every possible pair of means, while a Dunnett’s test was used to compare each group mean to a designated control mean. The Brown–Forsythe test was used to assess the assumption of equal variances prior to performing ANOVA. To compare cell viability at six and 24 h, two-way ANOVA analysis was performed. Statistical significance was defined as *p* ≤ 0.05.

## Results

### HM donor characteristics

Transitional HM from a total of 45 donors was collected and processed for EV isolation. An overview of the HM donor and infant characteristics can be found in Table S1. Per gestational age, 23 HM samples were obtained following preterm birth, and 22 following term birth. The infants’ gestational ages ranged from 23 to 41 weeks. In the total cohort, mothers gave birth to 28 (57.1%) male infants, while 21 (42.86%) were female. Four donors with twin births included both male and female infants, while three of the four twin births were preterm. The age of the HM donors ranged from 22 to 44 years, with a median age of 30 years (Table S1).

### HM EVs are enriched in immune-related surface markers

**Fig. 1:**
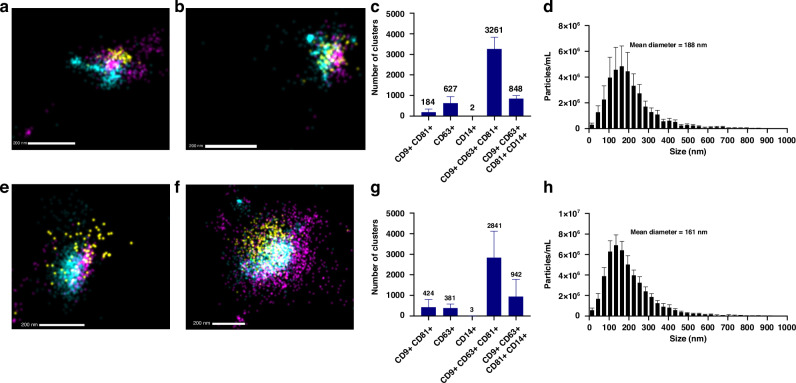
Characterization and super-resolution imaging of CD9, CD63, CD81, and CD14 HM EV surface markers. Term (**a**–**c**) and preterm (**e**–**g**) HM EVs are positive for CD14 (yellow); in addition to canonical EV markers CD63 (cyan), 81 and 9 (purple). The positive fluorescence signal per cluster of EVs did not differ significantly between term (**c**) and preterm (**g**) EVs. *n* = 2, term and preterm HM EVs. Histograms represent average from three fields of view per sample. **d**, **h** Particle size distribution measured using Zetaview. The mean EV diameter was 188 nm for term (**d**) and 161 nm (**h**) for preterm HM EVs, *n* = 8.

Following isolation, EVs were first characterized based on surface expression of canonical EV markers CD9, CD63, and CD81 using super-resolution imaging (Fig. 1). As expected, majority of the vesicles were triple positive for CD9, CD63, and CD81 surface expression (Fig. 1c, g). We have previously measured an enrichment in immune cell markers for preterm HM EVs, wherein CD14 was present in our proteomics analyses,^25^ thus CD14 surface expression was also explored on both term and preterm EVs (Fig. 1a–c, e–g). CD14 was detected in 848 out of 3261 vesicles in term HM EVs (Fig. 1c), and 942 out of 2841 vesicles in preterm EVs (Fig. 1g). Two term and three preterm HM EVs were single positive for CD14, indicating limited background signal and further confirming the presence of CD14 alongside CD9, CD63 and CD81 surface markers (Fig. 1c, g). Following particle size analysis to measure the size distribution of the EVs, the mean diameters for term and preterm HM EVs were 188 nm and 161 nm, respectively (Fig. 1d, h).

**Fig. 2:**
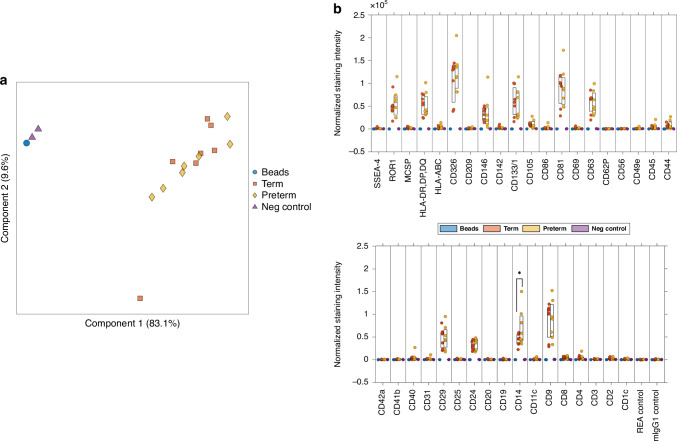
HM EV surface marker characterization. **a** Clustering of term and preterm HM EVs using principal component analysis (*n* = 7), negative control, and capture beads only control (*n* = 2). **b** Following background normalization, HM EVs expressed surface markers ROR1, HLA-DR,DP,DQ, HLA-ABC, CD326, CD146, CD133/1, CD105, CD86, CD45, CD44, CD40, CD29, CD24, CD14, and CD4, in addition to canonical EV markers CD9, 63 and 81. *n* = 2–7, *p < 0.04, Welch’s *t*-test.

We subsequently evaluated the CD14+ signal on term and preterm EVs, in a surface marker panel. Surface marker analysis was performed on term and preterm HM EVs using Miltenyi MACSPlex Exosome Kit, which measured fluorescence intensity of 37 surface epitopes using flow cytometry. Term and preterm EVs clustered together based on detectable surface markers (Fig. 2a). Besides the canonical EV markers CD9, CD63 and CD81, term or preterm HM EVs were positive for ROR1, HLA-DR, DP,DQ, HLA-ABC, CD326, CD146, CD133/1, CD105, CD86, CD45, CD44, CD40, CD29, CD24, CD14 and CD4 (Fig. 2b). A statistically significant difference between term and preterm samples was measured for CD14 only, where preterm had higher levels of CD14. No other significant differences among surface marker levels were detected.

### HM EVs induce protective signals and regulate inflammatory cytokines in human intestinal epithelial cells

**Fig. 3:**
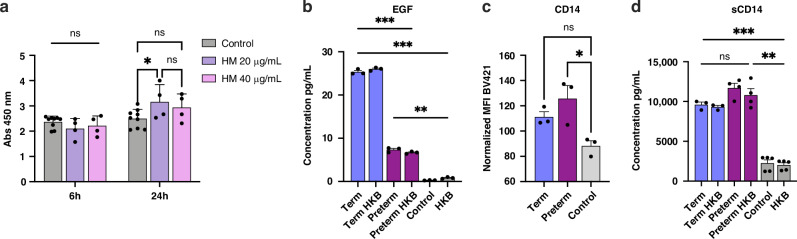
Effects of HM EVs on Caco-2 intestinal cells. **a** Cell viability following treatment with term HM EVs, or media only control for 6 or 24 h. Viability measured with XTT assay - absorbance (450 nm), *n* = 4–9, *p < 0.04, two-way ANOVA with Tukey’s test, two replicate experiments. **b** EGF levels in cell media following inflammatory activation with heat-killed bacteria (HKB, 10^9^ HKU) on cells treated for 22 h with term or preterm HM EVs (20 μg/ml), or media only control. Regardless of inflammatory activation with HKB, both term and preterm HM EVs significantly increased levels of EGF. Term HM EVs resulted in significantly higher levels when compared to preterm, *n* = 3. **c** CD14 surface expression normalized to HKB treatment, median fluorescence intensity (MFI) of BV421, *n* = 3, ns: *p* = 0.09. Plot is representative of two replicate experiments. **d** Soluble CD14 levels in media were significantly increased by term and preterm HM EVs, regardless of inflammatory activation with HKB, *n* = 3-4. ***p < 0.001, **p < 0.01, *p < 0.04, ns=non-significant, one-way ANOVA.

Confluent Caco-2 cell culture was used to model infant intestinal epithelium since Caco-2 cells 20-days post differentiation exhibit similar morphological and functional characteristics to human mid-gestation small intestinal villus enterocytes.^35,43,44^ When cells were treated with term HM EVs for 24 h, Caco-2 cell viability was increased at treatment concentrations of 20 µg/mL and 40 µg/mL. Statistical analysis using two-way ANOVA found an overall significant difference in viability across six and 24 h (DF_1,13_; *F* = 28.09; *p* < 0.04), and when combined with treatment (DF_2,13_; *F* = 6.458, *p* < 0.01), wherein post-hoc analysis revealed that treatment with 20 µg/mL of EVs significantly increased cell viability when compared to control at 24 h (Fig. 3a).

To further investigate if a protective effect of HM EVs on intestinal cells was induced, epidermal growth factor (EGF) levels in cell media were measured. Treatment of Caco-2 cells with term or preterm HM EVs significantly increased levels of EGF, regardless of inflammatory activation with heat-killed bacteria (Fig. 3b). Treatment with term HM EVs resulted in significantly higher EGF levels when compared to preterm HM EVs.

Since CD14 is proposed to facilitate homeostatic immune response in infants,^45^ and Caco-2 cells have been shown to express CD14, as well as release and take up soluble CD14 (sCD14),^46,47^ we sought to characterize CD14 levels in response to term or preterm HM EV treatment. Caco-2 surface expression of CD14 was analyzed using flow cytometry. Surface expression of CD14 was elevated following both term and preterm HM EV treatment, but significantly increased by preterm HM EVs (Fig. 3c). When soluble CD14 levels were measured in cell media, both term and preterm HM EVs significantly increased secretion of sCD14 regardless of inflammatory activation by heat-killed bacteria (Fig. 3d).

To investigate inflammatory markers on intestinal epithelial cells, a normal non-transformed cell line, human intestinal epithelial cells (HIECs), were used for further experiments. HIECs are normal embryonic human intestinal cells,^43^ which have been used to study endocytosis of HM EVs and miR cargo,^24,48^ and inflammatory response.^49–51^

**Fig. 4:**
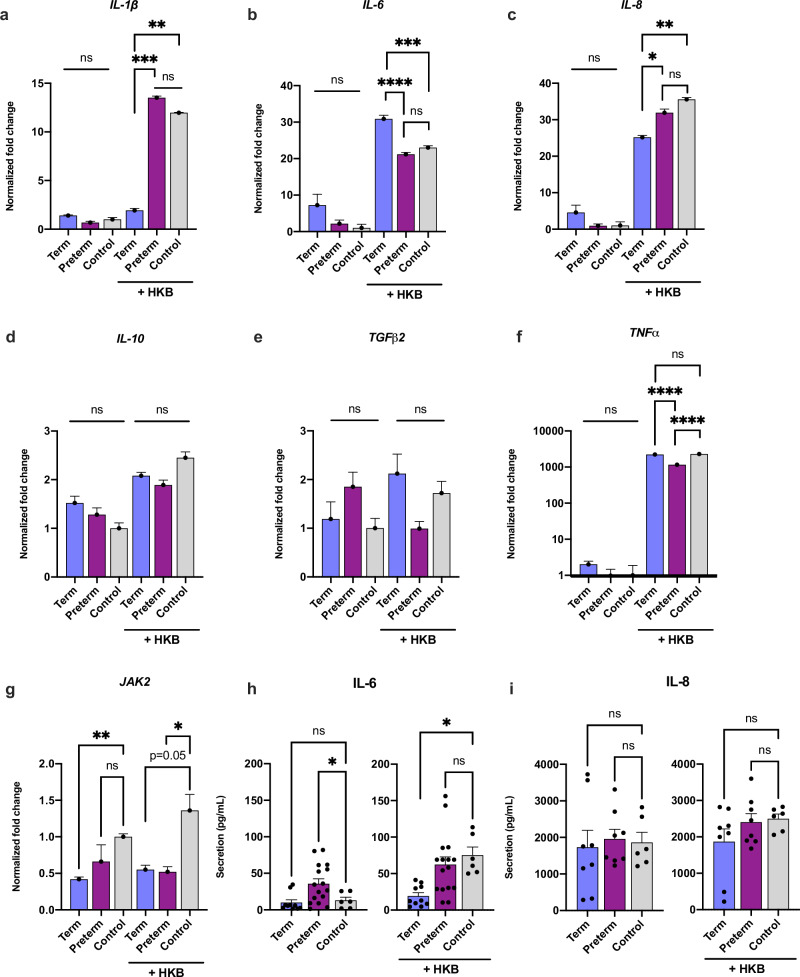
HM EVs regulate expression and secretion of cytokines and signaling proteins in human intestinal epithelial cells. Cells were treated with 20 µg/mL term or preterm HM EVs, followed by inflammatory activation with heat-killed bacteria (HKB, 10^9^ CFU). **a**–**g** Gene expression relative to no treatment control of media only (*y* = 1). **h**, **i** Secretion of IL-6 and IL-8 following treatment. *n* = 6–16, in replicate experiments. ****p < 0.0001, ***p < 0.001, **p < 0.01, *p < 0.04, ns=non-significant, one-way ANOVA with multiple comparisons.

Following inflammatory activation with heat-killed bacteria, pre-conditioning with term and preterm HM EVs reduced expression of IL-8 and TNFα (Fig. 4c, f). Term HM EVs decreased expression of IL-1β while increasing IL-6 expression. Conversely, preterm HM EVs increased IL-1β mRNA transcripts but had no significant effect on IL-6 expression (Fig. 4a, b). TGFβ2 and IL-10 expression was not significantly affected by either treatment (Fig. 4d, e). JAK2 expression was significantly reduced following term HM EV treatment alone. Following addition of heat-killed bacteria, both term and preterm HM EVs decreased JAK2 expression (Fig. 4g).

On the protein level, secretion of IL-6 and IL-8 in response to term or preterm HM EVs had significant differences between the two gestational ages (Fig. 4h). In the absence of heat-killed bacteria, term HM EVs had no effect on IL-6 levels in HIECs when compared to control, while preterm HM EVs significantly increased levels. When heat-killed bacteria were added, term HM EVs maintained IL-6 levels similar to control, while preterm HM EVs had no effect (Fig. 4h).

Though HM EVs affected IL-8 expression, the cytokine secretion in HIEC media was unaffected. Notably, the biological variance among individual HM EV donors was high (Fig. 4i). Overall, both term and preterm HM EVs modulated cytokines in HIECs, while only term samples downregulated IL-1β expression and IL-6 secretion.

### HM EVs downregulate inflammatory markers and cell death in human macrophages and monocytes

Prior to downstream analyses, HM EV uptake into THP-1 macrophages was visualized to determine a sufficient timeframe for EV uptake. HM EVs were taken up as early as 2 h post treatment when visualized with live cell imaging over the course of 4 h (Fig. S1a). Using confocal imaging, 6.5 h post treatment, EVs were taken up into endosomal and lysosomal compartments overlapping concomitantly with dextran on the intracellular periphery (Fig. S1b–e).

**Fig. 5:**
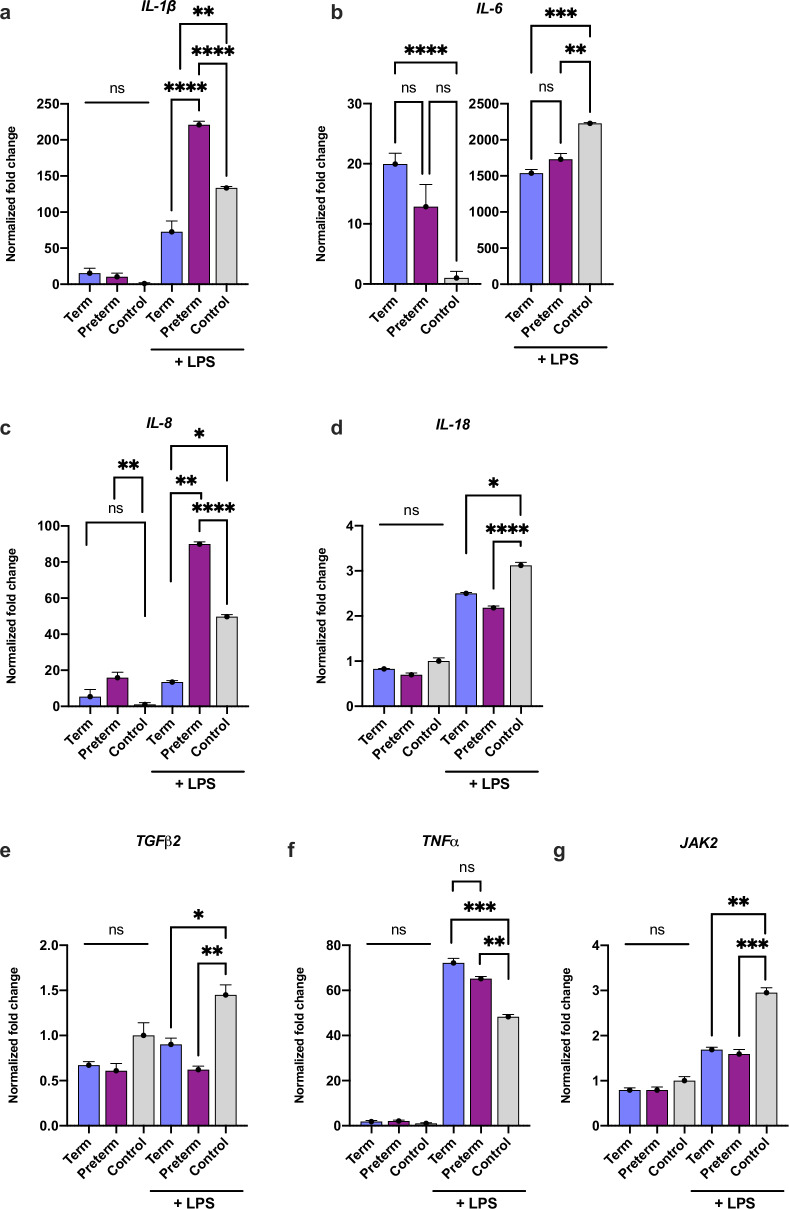
HM EVs regulate expression of cytokines and signaling proteins in macrophages. **a–g** Gene expression relative to no treatment control of media only (y = 1). Cells were pre-treated with 40 μg/mL term or preterm HM EVs, followed by inflammatory activation with LPS (1 ng/mL), in replicate experiments. ****p < 0.0001, ***p < 0.001, **p < 0.01, *p < 0.04, ns=non-significant, one-way ANOVA with multiple comparisons.

To test the effect of HM EVs on inflammatory regulation in immune cells, macrophages were pretreated with term or preterm HM EVs alone or followed by LPS for inflammatory activation. Treatment with LPS increased the expression of IL-1β, IL-6, IL-8, IL-18, and TNFα between two to 2000-fold in the macrophages (Fig. 5). In the presence of LPS, both term and preterm HM EVs significantly reduced the expression of IL-6, IL-18, and TGFβ2 (Fig. 5b, d, e). Preterm HM EVs upregulated expression of IL-1β and IL-8, while expression of the cytokines was significantly inhibited by term HM EVs (Fig. 5a, c). Expression of TNFα was significantly increased by both term and preterm HM EVs, when compared to control (Fig. 5f). In the absence of LPS, IL-6 expression was upregulated by term HM EVs, and IL-8 expression was significantly increased by preterm HM EVs (Fig. 5b, c). JAK2 expression was not significantly altered in control conditions, whereas following addition of LPS, both term and preterm HM EVs significantly reduced expression (Fig. 5g). In a cytometric bead array, secretion of IL-6, IL-8, IL-10 and TNFα was not significantly altered by HM EV pre-treatment (Fig. S2).

**Fig. 6:**
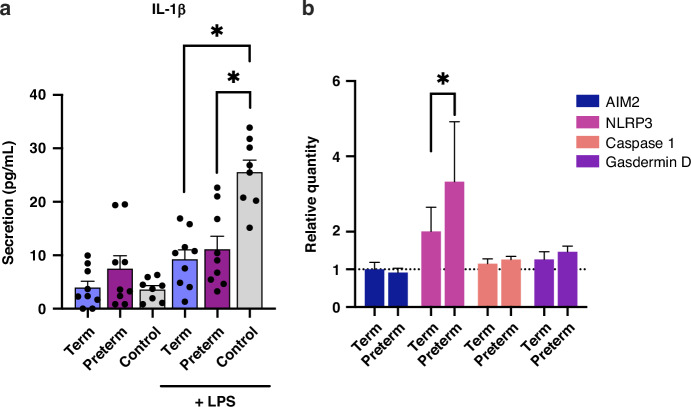
Term and preterm HM EVs regulate inflammasome in macrophages. **a** Secretion of inflammasome marker IL-1β was measured using ELISA in human PBMC-derived macrophages after pre-conditioning with 40 μg/mL preterm or term HM EVs for six hours, then inflammatory activation with LPS (1 ng/mL) for two hours, or media only control. *n* = 6–9, replicate experiments. *p < 0.04, one-way ANOVA. **b** Expression of NLRP3 inflammasome related genes NLRP3, AIM-2, Caspase 1, and Gasdermin D. Gene expression relative to LPS treatment. Gene expression below line at *y* = 1 on graph represents downregulation of expression. Treatment with both term and preterm EVs had limited effect on expression when compared to control for all genes except NLRP3. NLRP3 expression was significantly increased by preterm HM EVs when compared to term. *n* = 3, *p < 0.04, one-way ANOVA with multiple comparisons.

After measuring significant up- and downregulation of IL-1β expression, we measured secretion levels using ELISA. Following both term and preterm EV treatment and inflammatory activation, IL-1β secretion was significantly decreased (Fig. 6a). Macrophages can secrete IL-1β via a pattern recognition receptor known as NLRP3 (NOD-like receptor (NLR) family pyrin domain-containing 3), which leads to inflammasome activation.^14^ Since HM EVs regulated IL-1β expression and secretion, we explored whether the expression of proteins of the inflammasome complex were affected. Following treatment, gene expression of the inflammasome-associated AIM-2, Caspase-1, and Gasdermin D were generally unaffected by both term and preterm HM EVs, while NLRP3 expression was significantly increased. Notably, preterm HM EVs increased expression of NLRP3 more than term HM EVs (Fig. 6b).

**Fig. 7:**
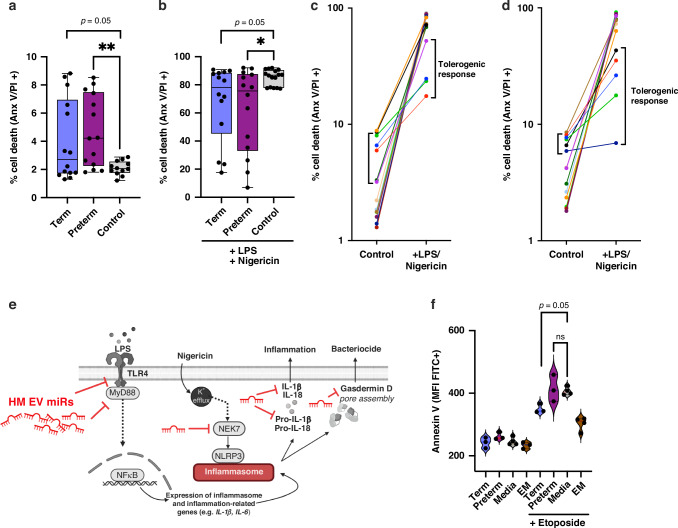
HM EVs regulate cell death in THP-1 monocytes. Cells were treated with 40 µg/mL term or preterm human milk EVs for 6 h, followed by inflammatory activation with 1 ng/mL LPS for two hours, and 10 µg/mL nigericin for 1 h. Cell death was measured in THP-1 cells treated with (**a**) term or preterm HM EVs, or media only control; (**b**) term or preterm HM EVs with LPS and nigericin, or LPS and nigericin alone. Protection against nigericin and LPS induced pyroptotic cell death was measured by flow cytometry of annexin V and propidium iodide signal. *n* = 10–16, four replicate experiments. Tolerogenic response was present in THP-1 cells treated with either term (**c**) or preterm (**d**) HM EVs. Tolerogenic response was proposed for biological replicates that increased cell death in control samples but protected against pyroptotic cell death following inflammatory activation, indicated in brackets. **e** HM EV miRs predicted targets TLR4, MYD88, and NEK7 could result in downregulation of programmed cell death via upstream inhibition of inflammasome pathway. **f** Term, but not preterm HM EVs, reduced apoptosis. THP-1 monocytes were treated with 40 µg/mL term or preterm human milk EVs for 6 h, followed by etoposide (30 µM) for 4 h, with or without an emricasan (EM, 10 µM) pre-treatment for 30 min, or media only control. Geometric mean of fluorescence intensity (FITC) was plotted, *n* = 3. **p < 0.01, *p < 0.04, ns=non-significant, one-way ANOVA with multiple comparisons.

Following effects on IL-1β and IL-18, both markers of the inflammasome, we sought to indirectly measure the effect of HM EVs on inflammasome-induced cell death. For an established and mechanistic analysis of inflammation in immune cells, the THP-1 human leukemia monocytic cell line was used.^52^ We followed a previously published method for priming THP-1 monocytes with LPS, followed by pyroptosis-induction using nigericin.^39^ We found that while HM EVs induced low levels of cell death when compared to media alone (Fig. 7a), they also significantly inhibited death induced by LPS and nigericin (Fig. 7b). Since we observed large biological variability in the HM EVs’ ability to attenuate cell death, we compared control and treated-groups for individual HM EV donor responses. Several samples that elicited an initial cell death response in the absence of inflammatory stimuli, also inhibited large-scale cell death when later treated with LPS and nigericin, suggesting the presence of a tolerogenic response. This effect was seen following treatment with both term and preterm HM EVs (Fig. 7c, d).

To gain insight into the HM EV cargo, by delineating which components may exert the cellular effects, we used Ingenuity Pathway Analysis (IPA, Qiagen) to investigate downstream targets of HM EV miRs. The most abundant term and preterm HM EV miRs, sequenced previously (Table S3), were predicted to directly target the IL-1 family, including IL-1β and IL-18, Gasdermin D, as well as members upstream of the inflammasome pathway: TLR4, MYD88, and NEK7^53^ (Figs. 7e, and S3, Table S4). Relevant for classical apoptotic cell death,^54^ HM EV miRs were also predicted to target Caspase-3 and -9, BCL-2, BAX, and BAK (Table S4). However, only term HM EVs exhibited protective effects in THP-1 monocytes against etoposide-induced apoptosis (Fig. 7f).

## Discussion

We found that both term and preterm HM EVs were enriched in epithelial (CD326),^55^ immune (HLA, CD24, CD14),^56–58^ and stem (CD133, CD29)^59,60^ cell-related surface markers (Fig. 2). Gestational-age based differences were detected for CD14, a modulator of toll-like receptor 2 and 4 signaling,^61^ which was more abundant on the surface of preterm HM EVs. In Caco-2 cells, treatment with term and preterm HM EVs upregulated CD14 surface expression, while secretion of soluble CD14 was increased following exposure to term and preterm HM EVs (Fig. 3c, d). Through cell surface activation and secretion, both the CD14 receptor and its soluble form promote immune tolerance, potentially contributing to the development of gut microbiome homeostasis.^62,63^

EVs from mature term HM have been shown to be enriched in CD14 compared to matched serum EVs, with CD326 and CD24 being the most abundant surface markers.^64^ In transitional HM EVs characterized here, CD326 was also among the most abundant. We did not detect CD3-positive vesicles, which have been previously measured on mature HM EVs.^64^ These differences could reflect changes in the EV-secreting cellular architecture as lactation progresses. Breast milk could contain higher concentrations of epithelial and immune cells.^65–68^ Here, increased CD14 levels on preterm HM EVs may indicate that immune cells are a major EV source. This finding is consistent with our previous work, which showed that preterm HM EVs have RNA signatures indicative of more abundant immune cell origins.^25^

In vulnerable infants with an immature intestine, the equipoise of intestinal epithelium and gut resident macrophages is paramount. Milk-derived EVs have been proposed to modulate immune cells associated with the oral and gut mucosa,^4,5^ while also supporting of epithelial cell function, which is crucial for maintaining intestinal homeostasis. EGF is present in HM and important in promoting the intestinal barrier.^69^ Term and preterm HM EVs significantly increased EGF levels (Fig. 3b), which could support the proliferation and differentiation of intestinal epithelial cells, and act as an anti-inflammatory mediator in the developing human intestine.^70^ We have previously identified abundant epidermal growth factor receptor (EGFR) kinase substrate 8-like protein 2 (EPS8), a regulator of EGFR signaling,^71^ from proteomics analysis of both term and preterm HM EVs cargo.^25^ EGFR may provide protection against NEC, supported by earlier research, where treatment with HM reduced TLR4 signaling and inhibited apoptosis via EGFR signaling.^72^

When investigating cytokine levels in the inflammatory response of human intestinal epithelial cells and PBMC-derived macrophages, term and preterm HM EVs had differential effects on the expression of cytokines IL-6, IL-8, and TNFα, influenced further by the cell type. For IL-6, epithelial cells showed an increase in expression in response to term HM EVs, with no significant change for preterm HM EVs (Fig. 4). We observed that in PBMC-derived macrophages, both HM EVs reduced expression of the cytokines (Fig. 5). In the intestine, IL-6 may have protective effects by supporting homeostasis by induction of cell proliferation, survival, and maintenance of crypt stem cells.^73^ Conversely, in macrophages, secretion of IL-6 indicates polarization to an inflammatory M1 phenotype, which is associated with NEC.^74,75^

Opposite expression patterns were seen for pro-inflammatory cytokines IL-8 and TNFα, with increases in macrophages (Fig. 5), but decreases in HIECs (Fig. 4). Preterm HM EVs were not able to attenuate IL-8 expression in HIECs, and increased it in macrophages, while term HM EVs significantly reduced its expression. Preterm neonates are more vulnerable to inflammatory diseases, and their immune system is still developing. In this context, preterm human milk EVs may play a role in boosting the immune system, preparing it to respond to potential infections and inflammation. By increasing IL-8 expression, preterm HM EVs may help recruit neutrophils to the site of inflammation, promoting a robust immune response, and enhancing the elimination of pathogens.^76^

Remarkably, regardless of cell type, term HM EVs downregulated IL-1β expression (Figs. 4 and 5), while both term and preterm significantly reduced their secretion (Fig. 6a). The decrease in LPS-associated cytokines, including IL-1β, following treatment with mature HM EVs has also recently been shown.^77,78^ Further evidence from mouse studies using bovine milk-derived EVs also indicate significant decreases in IL-1β.^5,79^ Taken together with our results, the downregulation of IL-1β appears to be conserved between different cell types – intestinal epithelial cells and macrophages tested here, and irrespective of lactation stage, or gestational age. IL-1β and IL-18 are potent pro-inflammatory cytokines that can promote polarization of T cells to an inflammatory phenotype.^80^ HM EVs ability to reduce cytokine levels in macrophages may support a more balanced and homeostatic immune response, aligning with previous findings in T cells.^4^

HM EVs protect against the development of NEC, potentially by inhibiting necroptosis, which has been partly attributed to HM oligosaccharides.^81^ We showed that term transitional HM EVs downregulated apoptosis in monocytes (Fig. 7f), and in earlier studies, cell death was reduced in intestinal epithelial cells in response to HM EV treatment.^82,83^ Here, EVs that increased cell death in the absence of LPS and nigericin, had an overall protective effect once pyroptosis was induced. This may indicate the promotion of tolerance to gut microbiota. While we detected a HM EV-dependent decrease in programmed cell death following treatment with inflammasome and pyroptosis inducer nigericin (Fig. 7), we measured a limited effect on Gasdermin D expression and an increase in NLRP3 expression (Fig. 6b). The selective regulation of the inflammasome components by term and preterm HM EVs may reflect a homeostatic regulation of the host immune response. The reduction in inflammatory cell death by HM EVs may support immune barrier function, allowing the macrophages to be retained, and to release Gasdermin D fragments thereby exerting cytotoxic effects on bacteria.^15^ Gasdermin D release from immune and intestinal epithelial cells has been shown to limit bacterial loads, specifically in the context of *Salmonella* infection.^84^ It could also stimulate goblet cells to secrete mucus, thus maintaining gut homeostasis.^85^

MiRs, such as miR-146a-5p and miR-148a, have been proposed to play a role in intestinal epithelium integrity and inflammasome regulation, which may be relevant in NEC-related signaling.^5,86,87^ Since biological variation in the abundance of miRs carried by HM EVs may influence their efficacy, we focused on the miRs that have been found to be most abundant. Based on our prior miR sequencing (Table S3), we propose that the downregulation of inflammatory cytokines and programmed cell death could be attributed to abundant miRs common to both term and preterm HM EVs, which also include miRs that are significantly downregulated in NEC.^88^ HM EV miRs were predicted to directly target IL-1 and IL-18, Gasdermin D, and upstream regulators of the inflammasome – NEK7, MYD88, and TLR4 (Fig. 7e).

For modulating the expression of cytokines, term and preterm miRs were enriched for targets in the JAK-STAT pathway. Indeed, following HM EV treatment, JAK2 expression was significantly downregulated in both macrophages and HIECs in the presence of inflammatory signals induced by LPS or heat-killed bacteria. JAK2 is also significantly upregulated in NEC intestinal tissue.^89^ MYD88 has been shown to activate JAK2 and STAT signaling, thereby miRs targeting both MYD88 and JAK2 may result in the downregulation of several key inflammatory genes, most notably, IL-6 and IL-1β.^90^ Thus, much of the cellular signaling and inflammatory response may be multi-targeted by HM EV miR cargo, with the abundant miRs conserved between HM donors (Table S4).

We observed large biological variability in the effects of HM EVs on cultured cells, indicating the need for large-scale studies. Nonetheless, our results suggest that supplementation with EVs from transitional HM may drive a tolerogenic immune response. Whether HM feeding itself, even when supplemented with a higher concentration of EVs, can effectively counteract bacterial infection, cytokine storm, and sepsis, remains to be elucidated. Since colostrum and transitional HM have been shown to contain a higher number of immunoregulatory proteins, miRs and EVs,^25,91–93^ supplementation strategies may have increased efficacy if derived from HM of those lactational stages. Interestingly, we found that only term HM EVs, not preterm, reduced IL-6 secretion in intestinal epithelial cells, attenuated expression of IL-1β and IL-8, and decreased apoptosis. Thus, term HM EV supplementation may also provide more effective protection for premature neonates, especially in their first few weeks of life, and could be preferential in compromised infants to prevent dysregulation of inflammatory signaling. Overall, these findings enhance our understanding of the bioactive components in transitional milk and could provide basis for both term and preterm HM EV supplementation in a clinical setting to investigate direct protection against NEC in targeted patient populations.^94,95^

## Supplementary information

Supplementary Table S3, S4 and Figure S3 Information

Supplementary Information

## Data Availability

Sequencing data discussed in this study is available in the supplementary materials.
