# Supplementary material for: Human milk extracellular vesicles modulate inflammation and cell survival in intestinal and immune cells
Source: Pediatr Res. 2024 Nov 28;98(1):314–26. doi: 10.1038/s41390-024-03757-5 (PMC12411258; doi:10.1038/s41390-024-03757-5)
Supplement: Supplementary file 2 — Supplementary Information [file 41390_2024_3757_MOESM2_ESM.pdf]

Table S1. Human milk donor information. Human milk was collected within two weeks following term or preterm birth. Infant's gestational age, age during the time of collection, sex, and birth weight, as well as mother's age was recorded.						
Donor ID	Infant's gestastional age	Term (T) or preterm (PT)	Infant's age (days)	Infant's sex	Infant's birth weight (grams)	Mother's age (years)
10	34 weeks, 4 days	PT	9	M	N/A	36
11	33 weeks, 5 days	PT	9	M	N/A	28
12	32 weeks, 2 days	PT	4	F	N/A	35
15	36 weeks, 5 days	PT	10	M	N/A	30
18	36 weeks, 4 days	PT	5	F	N/A	38
19	34 weeks, 3 days	PT	7	M	N/A	25
20	34 weeks, 1 day	PT	4	M	N/A	28
21	30 weeks, 5 days	PT	3	M	N/A	35
22	36 weeks, 2 days	PT	14	F	N/A	35
24	24 weeks, 3 days	PT	14	M	540	22
25	27 weeks, 1 day	PT	11	F	1167	30
27	29 weeks	PT	9	F	1370	26
28	28 weeks	PT	12	M and F	1080 and 1160	40
29	23 weeks, 6 days	PT	33	M	625	30
30	30 weeks, 1 day	PT	12	F	1430	30
31	34 weeks, 3 days	PT	5	M and F	2083 and 2226	33
32	29 weeks	PT	9	M	1050	26
33	34 weeks, 2 days	PT	8	M and F	2530 and 2410	29
34	35 weeks, 3 days	PT	11	M	2630	36
35	28 weeks, 4 days	PT	8	M	1281	44
36	29 weeks, 1 day	PT	7	M	1251	29
37	33 weeks, 2 days	PT	7	M	1895	30
38	27 weeks, 4 day	PT	12	F	1000	34
102	38 weeks, 3 days	T	10	M	N/A	31
107	39 weeks, 1 day	T	9	F	3540	34
110	41 weeks, 4 days	T	10	F	3291	30
111	40 weeks, 2 days	T	5	M	5868	25
112	39 weeks, 5 days	T	10	M	3061	32
113	40 weeks, 2 days	T	10	F	3517	24
114	38 weeks, 1 day	T	6	M	3911	33
115	38 weeks, 2 days	T	13	M and F	3316 and 3288	32
116	38 weeks	T	10	F	1595	31
117	40 weeks	T	11	F	3121	34
118	39 weeks, 6 days	T	8	M	2863	26
119	38 weeks, 3 days	T	13	M	2720	27
122	41 weeks, 0 days	T	5	F	3824	29
123	40 weeks, 2 days	T	5	M	3002	31
124	38 weeks, 4 days	T	5	F	2279	22
125	40 weeks	T	6	M	3569	28
126	40 weeks, 3 days	T	9	M	3487	27
129	38 weeks	T	6	M	3524	31
130	38 weeks	T	7	F	3022	23
131	38 weeks, 4 days	T	8	F	3536	39
133	41 weeks, 0 days	T	9	F	2811	31
136	37 weeks, 0 days	T	12	M	3002	30
					N/A - not available	

Table S2. Primer sequences for amplification of target genes using quantitative polymerase chain reaction.				
Gene	Forward primer	Reverse primer	Amplicon size	Source
AIM2	AGCCTGAACAGAAACAGATGG	CTTCTTGGGTCTCAAACGTGA	120	Liang, Ni, et al. "Overexpression of NLRP 3, NLRC 4 and AIM 2 inflammasomes and their priming-associated molecules (TLR 2, TLR 4, Dectin-1, Dectin-2 and NF κB) in Malassezia folliculitis." Mycoses 61.2 (2018): 111-118.
Beta-2-microglobulin	GGTTTCATCCATCCGACATTG	CATGTCTCGATCCCACTTAAC	212	Sun, Wenwen, et al. "Human epithelial-type ovarian tumour marker beta-2-microglobulin is regulated by the TGF-β signaling pathway." Journal of translational medicine 14 (2016): 1-13.
Caspase-1	TTTCCGCAAGGTTCGATTTTCA	GGCATCTGCGCTCTACCATC	54	Harvard primer bank
Gasdermin D	GGACAGGCAAAGATCGCAG	CACTCAGCGAGTACACATTCATT	76	Harvard primer bank
IL-10	TCCACGTGTTGAGATCATTGC	TCTTGATGGCCTTCGATTCTG	80	Sen, Ayantika, Anil Kaul, and Rashmi Kaul. "Estrogen receptors in human bladder cells regulate innate cytokine responses to differentially modulate uropathogenic E. coli colonization." Immunobiology 226.1 (2021): 152020.
IL-18	TCTTCATTGACCAAGGAAATCGG	TCCGGGGTGCATTATCTCTAC	75	Harvard primer bank
IL-1b	GAGCTCGCCAGTGAAATGATG	AGTGGTGGTCGGAGATTCGT	154	Hui, Ying, and Yan Yin. "MicroRNA-145 attenuates high glucose-induced oxidative stress and inflammation in retinal endothelial cells through regulating TLR4/NF-κB signaling." Life sciences 207 (2018): 212-218.
IL-6	GGTACATCCTCGACGGCATCT	GTGCCTCTTTGCTGCTTTCAC	81	Starkie, R. L., et al. "Carbohydrate ingestion attenuates the increase in plasma interleukin-6, but not skeletal muscle interleukin-6 mRNA, during exercise in humans." The Journal of physiology 533.2 (2001): 585-591.
IL-8	TTTTGCCAAGGAGTGCTAAAGA	AACCCTCTGCACCCAGTTTTC	194	Oglesby, Irene K., et al. "miR-17 overexpression in cystic fibrosis airway epithelial cells decreases interleukin-8 production." European Respiratory Journal 46.5 (2015): 1350-1360.
JAK2	CCAGATGGAAACTGTTCGCTCAG	GAGGTTGGTACATCAGAAACACC	126	Liao, Xiong-Yu, et al. "Ruxolitinib inhibits the proliferation and induces the apoptosis of MLL-r ALL cells through inactivating JAK/STAT signaling pathway." Translational Pediatrics 12.6 (2023): 1088.
NLRP3	GATCTTCGCTGCGATCAACAG	CGTGCATTATCTGAACCCCAC	81	Harvard primer bank
PPIA	GGCAAATGCTGGACCCAACACA	TGCTGGTCTTGCCATTCCTGGA	161	Primer3 (http://bioinfo.ut.ee/primer3)
RPLO	TCGACAATGGCAGCATCTAC	GCCTTGACCTTTTCAGCAAG	223	Primer3 (http://bioinfo.ut.ee/primer3)
TGFb2	CACGAACCCAAAGGGTACAA	ATATAAGCTCAGGACCCTGCT	96	Saleh, Mohamed Bou, et al. "Loss of hepatocyte identity following aberrant YAP activation: a key mechanism in alcoholic hepatitis." Journal of hepatology 75.4 (2021): 912-923.
TNFa	CCCAGGCAGTCAGATCATCTTC	GCTTGAGGGTTTGCTACAACATG	73	Dickel, Heinrich, et al. "Standardized tape stripping prior to patch testing induces upregulation of Hsp90, Hsp70, IL-33, TNF-α and IL-8/CXCL8 mRNA: new insights into the involvement of 'alarmins'." Contact dermatitis 63.4 (2010): 215-222.

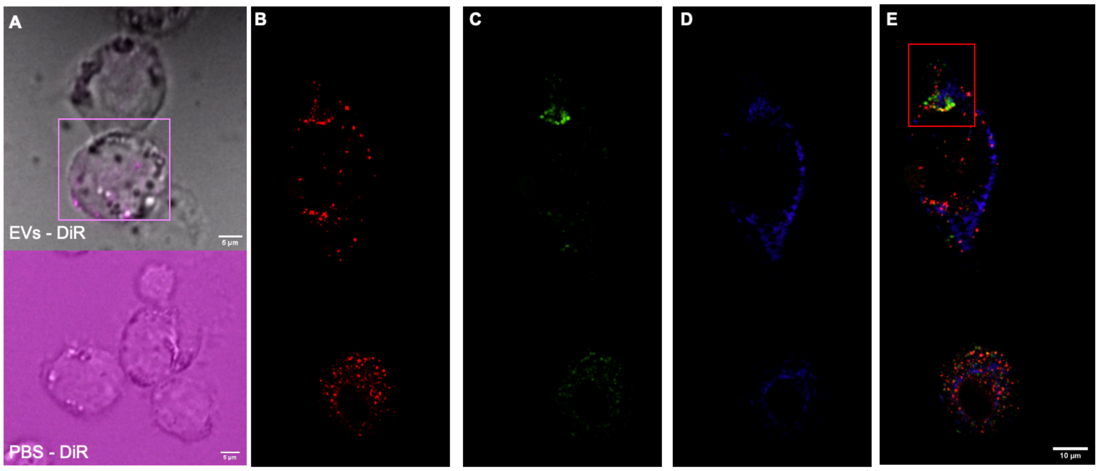

Figure S1. Uptake of HM EVs into THP-1 macrophages. (A) Cells were treated with 20µg/mL of DiR-labeled HM EVs (top panel) or DiR-PBS control (bottom panel). EV uptake was visualized in a time course of two to four hours post treatment. HM EV were taken up within four hours of treatment (pink box). **(B-E)** Cells were treated with 20µg/mL of DiR-labeled HM EVs (red) for five hours, followed by one hour of 10kDa dextran (**C**, green - FITC, 0.5mg/mL), and WGA for 30 minutes to stain the cell membrane (**D**, blue – AF350, 50µg/mL). EV uptake and dextran co-localization were visualized seven hours post treatment (**E**, red box). Z-stack shown from middle of cell.

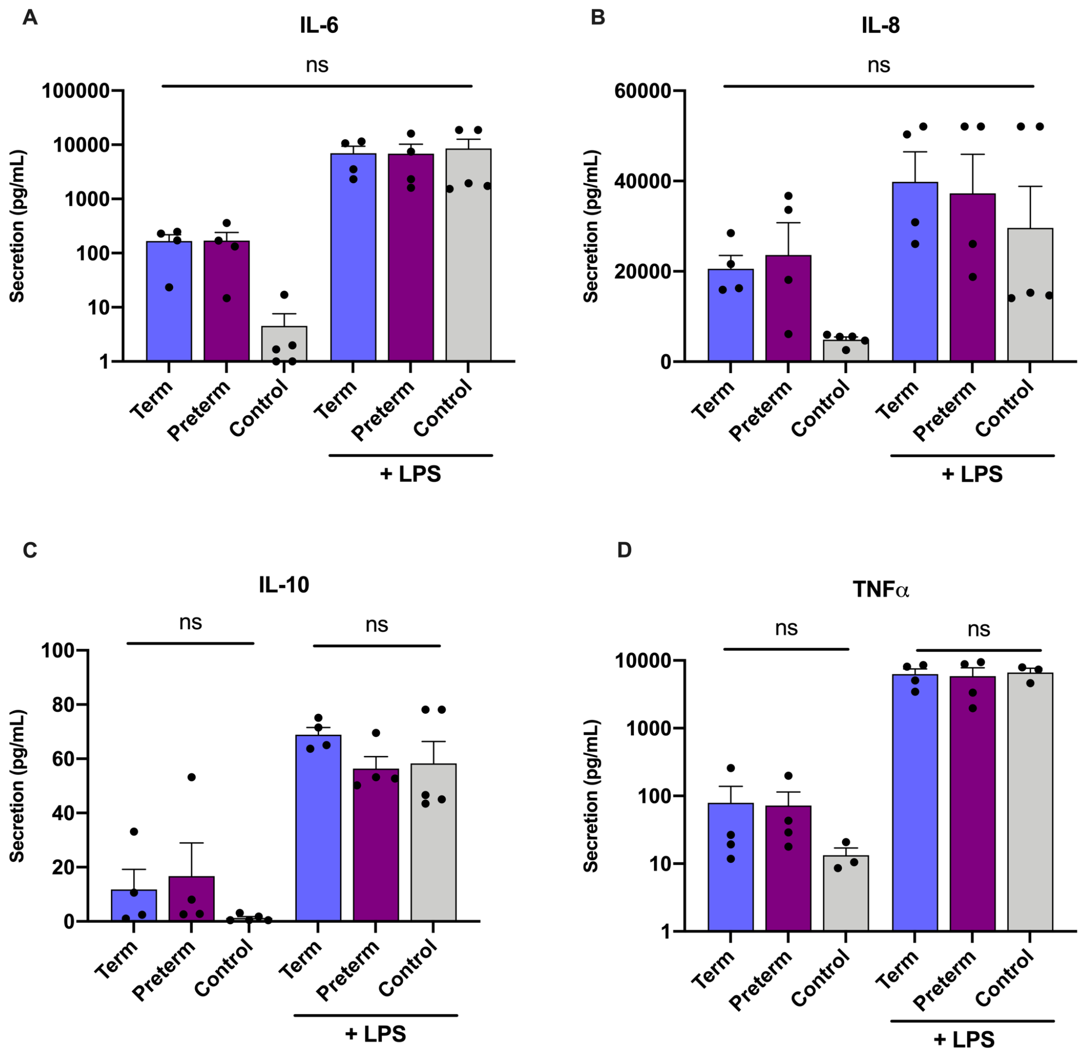

Figure S2. Secretion of cytokines in macrophages. PBMC-derived macrophages were pre-treated with 40μg/mL term or preterm HM EVs, followed by inflammatory activation with LPS (1ng/mL) for two hours. Secretion relative to LPS treatment control was measured using cytometric bead array. n=4-5, two replicate experiments. ns=non-significant, one-way ANOVA with multiple comparisons.
